# Supplementary material for: The Asian tiger mosquito Aedes albopictus (Skuse) in Kosovo: First record
Source: PLoS One. 2022 Mar 15;17(3):e0264300. doi: 10.1371/journal.pone.0264300 (PMC8923454; doi:10.1371/journal.pone.0264300)
Supplement: Attachment — Submitted filename: PONE-D-21-33477_reviewer 3.pdf [file pone.0264300.s002.pdf]

PLOS ONE

The Asian tiger mosquito Aedes albopictus (Skuse) in Kosovo: first record

--Manuscript Draft--

**Manuscript Number:**	PONE-D-21-33477
**Article Type:**	Research Article
**Full Title:**	The Asian tiger mosquito Aedes albopictus (Skuse) in Kosovo: first record
**Short Title:**	Aedes albopictus in Kosovo: first record
**Corresponding Author:**	Enkelejda Velo, PhD<br>Institute of Public Health<br>Tirana, ALBANIA
**Keywords:**	Keywords: Aedes albopictus, tiger mosquito, invasive species.
**Abstract:**	The Asian tiger mosquito, Aedes albopictus , is an invasive mosquito species that is considered a potential vector of about 22 arboviruses, among which dengue, chikungunya and Zika. Despite the investigations carried out for this species' presence in Kosovo since 2017 under the VectorNet project framework, there has been no finding of Ae. albopictus . Here we report the first detection of Aedes albopictus on the territory of the Republic of Kosovo. The first finding in July 2020 was driven by a photo of adult mosquito published in social media by a citizen in one of the villages were the surveillance was ongoing. The subsequent field investigation in July 2020 confirmed the presence of adult mosquitoes by human landing catch and collection of eggs in ovitraps at the village of Zhur. Ovitraps with seed germination paper were deployed at the ground crossing Morina, at Pand and several villages near the border with Albania, and more apart from border crossings in Prizren. Monitoring was performed at 10 stations, 37 sampling stations in the Northern part of Kosovo, for 7 weeks with ovitraps and BG-Sentinel. Fifty-two out of 81 ovitraps were positive for the presence of Ae. albopictus . A total of 2711 eggs were collected. The citizen science platform Mosquito Alert (AIM Cost action) were presented to the local community to raised local participation and to have more evidence from the other areas. The evidence of the first finding of the Asian tiger mosquito is significant regarding public health.
**Order of Authors:**	Nesade Muja-Bajraktari, PhD
	Përparim Kadriaj, PhD
	Ferdije Zhushi-Etemi, Prof.
	Kurtesh Sherifi, Prof.
	Bulent Alten, Prof.
	Dusan Petrić, Prof.
	Enkelejda Velo, PhD
	Francis Schaffner, PhD
**Additional Information:**	
**Question**	**Response**
**Financial Disclosure**<br><br>Enter a financial disclosure statement that describes the sources of funding for the work included in this submission. Review the submission guidelines for detailed requirements. View published research articles from *PLOS ONE* for specific examples.	The author(s) received no specific funding for this work.

Powered by Editorial Manager® and ProduXion Manager® from Aries Systems Corporation

This statement is required for submission and **will appear in the published article** if the submission is accepted. Please make sure it is accurate.<br><br>**Unfunded studies**<br>Enter: *The author(s) received no specific funding for this work.*<br><br>**Funded studies**<br>Enter a statement with the following details:<br>• Initials of the authors who received each award<br>• Grant numbers awarded to each author<br>• The full name of each funder<br>• URL of each funder website<br>• Did the sponsors or funders play any role in the study design, data collection and analysis, decision to publish, or preparation of the manuscript?<br>• **NO** - Include this sentence at the end of your statement: *The funders had no role in study design, data collection and analysis, decision to publish, or preparation of the manuscript.*<br>• **YES** - Specify the role(s) played.<br><br>* typeset	
**Competing Interests**<br><br>Use the instructions below to enter a competing interest statement for this submission. On behalf of all authors, disclose any competing interests that could be perceived to bias this work—acknowledging all financial support and any other relevant financial or non-financial competing interests.<br><br>This statement is required for submission and **will appear in the published article** if the submission is accepted. Please make sure it is accurate and that any funding sources listed in your Funding Information later in the submission form are also declared in your Financial Disclosure statement.<br><br>View published research articles from	The authors have declared that no competing interests exist.

PLOS ONE for specific examples.<br><br>**NO authors have competing interests**<br><br>Enter: *The authors have declared that no competing interests exist.*<br><br>**Authors with competing interests**<br><br>Enter competing interest details beginning with this statement:<br><br>*I have read the journal's policy and the authors of this manuscript have the following competing interests: [insert competing interests here]*<br><br>* typeset	
**Ethics Statement**<br><br>Enter an ethics statement for this submission. This statement is required if the study involved:<br><br>• Human participants<br>• Human specimens or tissue<br>• Vertebrate animals or cephalopods<br>• Vertebrate embryos or tissues<br>• Field research<br><br>Write "N/A" if the submission does not require an ethics statement.<br><br>General guidance is provided below. Consult the submission guidelines for detailed instructions. **Make sure that all information entered here is included in the Methods section of the manuscript.**	N/A

**Format for specific study types**<br><br>**Human Subject Research (involving human participants and/or tissue)**<br>• Give the name of the institutional review board or ethics committee that approved the study<br>• Include the approval number and/or a statement indicating approval of this research<br>• Indicate the form of consent obtained (written/oral) or the reason that consent was not obtained (e.g. the data were analyzed anonymously)<br><br>**Animal Research (involving vertebrate animals, embryos or tissues)**<br>• Provide the name of the Institutional Animal Care and Use Committee (IACUC) or other relevant ethics board that reviewed the study protocol, and indicate whether they approved this research or granted a formal waiver of ethical approval<br>• Include an approval number if one was obtained<br>• If the study involved *non-human primates*, add *additional details* about animal welfare and steps taken to ameliorate suffering<br>• If anesthesia, euthanasia, or any kind of animal sacrifice is part of the study, include briefly which substances and/or methods were applied<br><br>**Field Research**<br><br>Include the following details if this study involves the collection of plant, animal, or other materials from a natural setting:<br>• Field permit number<br>• Name of the institution or relevant body that granted permission	
**Data Availability**<br><br>Authors are required to make all data underlying the findings described fully available, without restriction, and from the time of publication. PLOS allows rare exceptions to address legal and ethical concerns. See the PLOS Data Policy and FAQ for detailed information.	Yes - all data are fully available without restriction

A Data Availability Statement describing where the data can be found is required at submission. Your answers to this question constitute the Data Availability Statement and **will be published in the article**, if accepted.<br><br>**Important:** Stating 'data available on request from the author' is not sufficient. If your data are only available upon request, select 'No' for the first question and explain your exceptional situation in the text box.<br><br>Do the authors confirm that all data underlying the findings described in their manuscript are fully available without restriction?	
**Describe where the data may be found in full sentences. If you are copying our sample text, replace any instances of XXX with the appropriate details.**<br><br>• If the data are **held or will be held in a public repository**, include URLs, accession numbers or DOIs. If this information will only be available after acceptance, indicate this by ticking the box below. For example: *All XXX files are available from the XXX database (accession number(s) XXX, XXX.).*<br>• If the data are all contained **within the manuscript and/or Supporting Information files**, enter the following: *All relevant data are within the manuscript and its Supporting Information files.*<br>• If neither of these applies but you are able to provide **details of access elsewhere**, with or without limitations, please do so. For example:<br><br>*Data cannot be shared publicly because of [XXX]. Data are available from the XXX Institutional Data Access / Ethics Committee (contact via XXX) for researchers who meet the criteria for access to confidential data.*<br><br>*The data underlying the results presented in the study are available from (include the name of the third party*	All relevant data are within the manuscript and its Supporting Information files.

and contact information or URL).<br>• This text is appropriate if the data are owned by a third party and authors do not have permission to share the data.<br><br>* typeset	
Additional data availability information:	

Manuscript Click here to access/download;Manuscript;Manuscript.docx

The Asian tiger mosquito *Aedes albopictus* (Skuse) in Kosovo: first record

Nesade Muja-Bajraktari[1], Përparim Kadriaj[2], Ferdije Zhushi-Etemi[1], Kurtesh Sherifi[3], Bulent Alten[4], Dusan Petrić[5], Enkelejda Velo[2]*, Francis Schaffner[6]

[1] *Department of Biology, Faculty of Mathematics and Natural Science, University" Hasan Prishtina", Prishtina, Republic of Kosovo.*

[2] *Control of Infectious Diseases Department, Institute of Public Health, Tirana, Albania.*

[3] *Department of Veterinary Medicine, Faculty of Agriculture and Veterinary, University" Hasan Prishtina", Prishtina, Republic of Kosovo.*

[4] *Hacettepe University, Faculty of Science, Biology Department, Ecology Division, VERG Laboratories, Ankara, Turkey.*

[5] *Laboratory for medical and veterinary entomology, Faculty of Agriculture, University of Novi Sad, Novi Sad, Serbia.*

[6] *Francis Schaffner Consultancy, Riehen, Switzerland.*

*Corresponding author

E-mail: *keladikolli@yahoo.com* (EV)

Abstract

The Asian tiger mosquito, *Aedes albopictus*, is an invasive mosquito species that is considered a potential vector of about 22 arboviruses, among which dengue, chikungunya and Zika. Despite the investigations carried out for this species' presence in Kosovo since 2017 under the VectorNet project framework, there has been no finding of *Ae. albopictus*. Here we report the first detection of *Aedes albopictus* on the territory of the Republic of Kosovo. The first finding in July 2020 was driven by a photo of adult mosquito published in social media by a citizen in one of the villages were the surveillance was ongoing. The subsequent field investigation in July 2020 confirmed the presence of adult mosquitoes by human landing catch and collection of eggs in ovitraps at the village of Zhur. Ovitraps with seed germination paper were deployed at the ground crossing Morina, at Pand and several villages near the border with Albania, and more apart from border crossings in Prizren. Monitoring was performed at 10 stations, 37 sampling stations in the Northern part of Kosovo, for 7 weeks with ovitraps and BG-Sentinel. Fifty-two out of 81 ovitraps were positive for the presence of *Ae. albopictus*. A total of 2711 eggs were collected. The citizen science platform Mosquito Alert (AIM Cost action) were presented to the local community to raised local participation and to have more evidence from the other areas. The evidence of the first finding of the Asian tiger mosquito is significant regarding public health.

Keywords: *Aedes albopictus*, tiger mosquito, invasive species.

Introduction

Aedes (*Stegomyia*) *albopictus* (Skuse, 1894) (Diptera: Culicidae), commonly called the 'Asian tiger mosquito', is widespread throughout the tropical and subtropical regions of the world, partly also in the Mediterranean area. Many countries worldwide have been invaded by this species in last 30 years, including various regions in America and the Mediterranean.

It is currently considered one of the top 100 invasive species globally and the most invasive mosquito species [1], [2] . It is of medical importance due to its aggressive daytime human-biting behavior and vectorial competence being considered a severe threat to human health because of its potential to transmit 22 arboviruses in the family Flaviviridae (e.g., dengue, West Nile, yellow fever, Japanese encephalitis), Bunyaviridae (e.g., Rift Valley fever, Potosi, Cache Valley, and LaCrosse viruses), Togaviridae (e.g., chikungunya and Ross River virus) [3], [4], [5], [6], [7]. After *Ae. aegypti*, *Ae. albopictus* is the secondary vector of dengue and dengue hemorrhagic fever [8]. In Europe, the species acts as a dengue vector and was incriminated as the only vector in the first European outbreak of chikungunya in northeastern Italy [9], dengue case in 2010 in Croatia [10], Zika and dengue cases in 2019 in France [11] ,[12], [13].

The first report of the tiger mosquito in the European continent was recorded in 1979 in Albania from Adhami and Murati 1987 [14]. It is thought to have been imported in shipments and containers from China in the mid-1970s (1975). Today, it is still distributed throughout the country [15], unfortunately is not banned only in Albania, but it is spread throughout the European continent with different ways. According to the existing data, this species was recorded in Montenegro [16], but there is no clear evidence either from neighboring Albania or from Italy. Actually, it was found in a used tire imported from Germany, in North Macedonia [17], and Serbia [18]. In Serbia, *Ae. albopictus* was intercepted in two districts in the western and southwestern part of the country. It has been present for the past nine years on the Croatian border (Batrovci, northwest of Serbia), [19] and on the Montenegro border (Dobrakovo, southwest of Serbia) since 2014 [20] .

Distribution models predict that *Ae. albopictus* will continue to expand depending on transport, environmental, and climatic changes [21], [22], [23].

This worldwide expansion is mostly due to dormant egg transport via the international trade in used tires [24] and shipments of the Asian plant ‘‘lucky bamboo’’ (Dracaena spp.) [25], [26] and

by public and private ground transport from heavily infested areas [23]. In 2017, a research for tiger mosquito in Kosovo was conducted at the borders with Macedonia and Albania within the VectorNet project framework. It resulted negative, although Kosovo has favorable conditions for the development of mosquito's species [27].

The primary objective of this study was to verify the tiger mosquito's presence in the territory of Kosovo and monitor its distribution. Here we report the first detection of *Ae. albopictus* in Kosovo.

Materials and Methods

Study area

The present study was conducted in the municipality of Prizren (42.121664 ˚N, 20.733329˚ E) and in Suhareka city (42.2248 ˚N, 20.2248˚N) in July, August and September 2020. The Municipality of Prizren, respectively South Dukagjini, occupies the southern position in the Dukagjini Plain and southwestern Kosovo. The average altitude is about 450 m above sea level and includes nearly 640 km^2 of Kosovo's entire surface. Sharri mountain, Prizren plain and downstream of the Drini i Bardhe define the relief. Sharri mountain is also an essential geographical element, constitutes a watershed between the Adriatic Sea and the Aegean Sea basin. The main aspects of the climate are temperatures, precipitation and winds. Where based on these elements, the climate of this part of Kosovo is the Mediterranean. The city of Suhareka is located in the southern part of Kosovo. The average altitude is about 455 m. It is characterized by the developed hydrographic chain. Respectively there are a large number of rivers that pass through its territory.

Collection and identification

We used ovitraps considered the best method for detecting the females' presence via egg laying [28] and entomological aspirator (Genicco Srl,Item, Model: JF0825S1H—R) and BG-Sentinel traps, (https.//eu.biogents.com/wp-content/uploads/BG-Sentinel-2-Manual-EN-web.pdf), to collect the adult mosquitoes. The ovitraps (500 ml black plastic cups) filled with tap water and a masonite strip (12.5 × 2.5 cm) and filter paper (38 x 9 cm) for egg deposition were used. The

plastic cups were modified by two holes punching 3 cm from the top of the cup to prevent water overfilling. A total of 81 ovitraps were randomly distributed to 37 sampling stations (4-28 ovitraps) in 10 localities [Vërmicë (1), Zhur (3), Vlashnje (1), Prizren (2), Atmaxh (1), Landovicë (1) and Suhareka (1)]. The aerial distance between the traps was minimum 100 m. The ovitraps were placed on the ground, in shaded and accessible places, under vegetation, with free space above at least 1 m, (Figure 1. a, b, c, d).

Fig 1. Examples of traps operating at several mosquito collection sites.

Ovitraps in: a Vlashnje (Tyre centres); b Zhur st.1 (Privat residence garden); c Prizren (Privat residence garden); d Vërmicë (Restaurant veranda); e Odour-baited adult traps (BG-Sentinel) Vermice (Restaurant garden); f Zhur st.1 (Resident garden); g Prizren (Resident garden). Catching with aspirator: h Vlashnje (Tyre centres). Adults resting in: i Prizren (on the human body); j Zhur (Plastic bottle); k Vlashnje (Inside the tire, resting on the surface water); l Zhur st.1 (First specimen of *Aedes albopictus* resting in the human body).

The ovitraps were left in the same place during the monitoring (10 days), then the filter paper and masonite strips were collected from the sampling stations and were transferred to the laboratory in Prishtina University, for identification. After having adults in the laboratory, the adult mosquitoes were identified using the MosKeyTool [29]. Another technique used in this research was aspiration from human bite for 30 minutes. This technique from human bite it was used in three localitities, (Zhur st.1, Vlashnje, Prizren, (Table 1) for 30 minutes, and two volunteers were active for three entomological survive period (Fig 1 h, i, l).

Table 1 Results of *Ae. albopictus* adult trapping with BG sentinel traps (BG) or handheld electric aspirators (A) on the Prizreni municipality (July -September 2020).

Locality	Latitude (N)	Longitude (E)	Method of capture	Sampling area	Sampling period#	No. of adults	No. of adults
						Ae. albopictus	*Cx.pipiens s.l*

Vërmicë	42.166918	20.572473	BG	Restaurant garden with vegetations	25-07-20<br>09-08-20<br>25-08-20	0<br>15<br>12	7<br>3<br>3
Zhur st.1	42.16604	20.61539	BG	Private residence (garden)	25-07-20<br>09-08-20<br>25-08-20	27<br>10<br>5	3<br>0<br>0
Zhur st.2	42.161245	20.623351	BG	Private residence (garden)	25-07-20<br>09-08-20<br>25-08-20	0<br>4<br>0	2<br>5<br>0
Prizren	42.223997	20.734394	BG	Private residence (garden	25-07-20<br>09-08-20<br>25-08-20	0<br>2<br>3	2<br>5<br>7
Zhur st.1	42.168164	20.615606	A	in the yard of house	01-08-20<br>10-08-20	1<br>3	0<br>0
Vlashnje	42.198731	20.667758	A	tire centers	03-09-20	9	0
Prizren	42.223997	20.734394	A	in the house with vegetable garden	30-08-20<br>08-09-20	1<br>1	0<br>0

[#]Entomological survey period: I: 25–27 July 2020; II: 08–10 August 2020; III: 25–27 August 2020

In four localities (Vërmicë, Zhur st.1, Zhur st.2, Prizren) are used BG-Sentinel trap baited with BG lure and CO_2 (https://eu.biogents.com/wp-content/uploads/BG-Sentinel-2-Manual-EN-web.pdf). One BG-Sentinel trap was running for two consecutive nights every two weeks for three entomological survive periods (Table 1). The traps were set in the private houses' backyard (Fig 1. e, f, g). In total during this research are used 12 BG-Sentinel traps.

Results

The presence of *Ae. albopictus* is registered in Kosovo for the first time at the end of July 2020 in Zhur village. The annoyance and bites that this strange black and white mosquito had caused to citizens during this period was why the citizens had reacted and photographed it. This information alarmed us that the tiger mosquito could be present in this area. After we captured

the first specimen and identified it in the laboratory in the Institute of Public Health in Tirana, we concluded that it is *Ae. albopictus*. It was a male mosquito (Fig 2) found in a private residence garden, and it was caught by hand.

Fig 2. First specimen under the stereomicroscope (N.Muja-Bajraktari)

In total, 2711 eggs were collected during this investigation in ten localities in the Prizreni Region with 37 sampling stations (Fig 3).

Fig 3. Distribution map of the ovitraps in Prizreni Region

Table 1 shows placement of ovitraps and numbers of eggs collected on the Prizreni municipality. Out of 14 from 37 sampling stations, no eggs were collected in the study area. We counted 440 eggs in Vërmicë, 1187 in Zhur, 139 in Vlashnje and 119 in Atmaxha, respectively.

In the city of Prizren we collected 786 eggs in three stations, while 40 eggs were counted in the second locality in Truck Terminal, the periphery of the city of Prizren. We didn't have any egg in the Landovica and Suhareka city (Table 2).

Table 2. Placement of ovitraps and numbers of eggs collected on the Prizreni municipality, with the respective identification number (ID), georeferenced, sampling area, entomological survey and number of *Ae. albopictus* eggs found.

Localities	ID	Sampling period[#]	Latitude (N)	Longitude (E)	Sampling area	No.of eggs	
						Ae. albopictus	*Ae. geniculatus*
Vërmicë	01/1	I, II, III	42.167278	20.577672	Near the road	68	63
	01/2	I, II, III	42.168801	20.582681	Near the road	54	0
	01/3	I, II, III	42.166918	20.572473	Restaurant garden	202	0
	01/4	I, II, III	42.164574	20.567957	Restaurant	112	0
	01/5	I, II, III	42.163792	20.563543	(veranda)	4	0

Total						440	63
Zhur	02/1	I, II, III	42.168164	20.615606	Private residence (garden)	346	0
	02/2	I, II, III	42.166543	20.607746	Private residence (garden with pets)	196	0
	02/3	I, II, III	42.161062	20.622807	Private residence (garden)	168	48
	02/4	I, II, III	42.15736	20.618477	Private residence (garden)	62	0
	03/1	I, II, III	42.165116	20.623798	Private residence (garden with vegetation)	175	0
	03/2	I, II, III	42.161867	20.611347	Private residence (garden)	240	0
	03/3	I, II, III	42.223801	20.734074	Private residence (garden)	0	0
	03/4	I, II, III	42.15956	20.629809	Private residence (garden)	0	0
	04/1	IV	42.163807	20.632446	Private residence (garden with chicken)	0	0
	04/2	IV	42.166677	20.627916	Private residence (garden)	0	0
	04/3	IV	42.15726	20.624888	Near the forest	0	0
Total						1187	48
Vlashnje	05/1	II, III	42.200968	20.660929	At the gas station near the road	76	0
	05/2	II, III	42.204696	20.659614	Inside the tires	30	0
	05/3	II, III	42.201094	20.664804	Inside the tires	33	0
	05/4	II, III	42.203438	20.668572	Tire centers (garden)	0	0
	05/5	II, III	42.200968	20.660929	Tire centers (garden)	0	0
Total						139	0
Prizren	06/1	I, II, III, IV	42.223288	20.741278	Private residence (garden)	438	4
	06/2	I, II, III	42.228297	20.739493	Private residence (garden)	176	0
	06/3	I, II, III	42.226967	20.729043	Private residence (garden)	172	0
	06/4	I, II, III	42.223801	20.734074	Private residence (garden)	0	0
Total						786	4
Truck Terminal	07/1	IV	42.249954	20.729769	Truck terminal(garden)	3	0
	07/2	IV	42.256972	20.733839	Restaurant(garden)	16	0

	07/3	IV	42.254285	20.72666	Near the road	0	0
	07/4	IV	42.250268	20.721548	Truck terminal (garden)	21	0
Total						40	0
	08/1	III	42.245329	20.699713	At the gas station near the road	73	0
Atmaxhë	08/2	III	42.24801	20.696709	Near the car wash	46	0
	08/3	III	42.243715	20.702604	Hotel garden	0	0
Total						119	0
	09/1	IV	42.259853	20.688001	Supermarket forecourt	0	0
Landovicë	09/2	IV	42.264082	20.6842	Near the road	0	0
	09/3	IV	42.255862	20.684525	Privat residence(garden)	0	0
Suharekë	10/1	I, II, III, IV	42.363973	20.832803	Bus station	0	0
Overtotal						2711	115

#Entomological survey period: I: 23–02 July 2020; II: 02–12 August 2020; III: 12–22 August 2020; IV: 02–12 September 2020

Also, four adult female were collected with an aspirator in a yard of house nearby resident, nine adult mosquitoes were also collected with aspirator in tire centers where there was a lot of water accumulated from atmospheric precipitation through the tires, (Figure 1 h, k) and two males were collected in the house with a vegetable garden. We collected 78 adult mosquitoes at four sampling stations in the gardens with vegetation of residential houses and in a restaurant's garden for three entomological survey periods with the BG-Sentinel trap. Among them, 38 were female and 39 males (Table 1).

Forty-eight eggs were successfully hatched and were reared to adults (18 male/30 female). All adult mosquitoes were morphologically identified and classified as *Ae. albopictus*. During the research period, we also identified 115 eggs of *Ae. geniculatus* caught with ovitraps (Table 2) and 34 adult mosquitoes of *Cx. pipiens s.l*. caught with BG-Sentinel trap. (Table 1).

Discussion

The "Asian tiger mosquito", *Ae. albopictus*, originating from Southeast Asia, has undergone a significant expansion of its range in the last few decades [30].

In Europe, the tiger mosquito was first reported in Albania in 1979 [14], than ten years later in Italy [31], France [32], [33], Spain [34], Belgium [35], Switzerland [36], Greece [37], Montenegro [18],[38], Croatia [16], Bosnia and Herzegovina [16], Slovenia [16], [39] and North Macedonia [17]. As in other cases [40], [41], it is challenging to speculate when the Tiger mosquito arrived in these parts of Kosovo, the mosquito had never been reported there before, probably because of the low density of *Ae. albopictus* populations did not create a nuisance for the inhabitants, who did not notice its presence. The maritime route is the most likely pathway, given the high percentage of containers and goods transported by ship. The movement of cars has helped a lot in distributing *Aedes albopictus* species [42]. The first identification of the tiger mosquito was made near the border with Albania. It is thought that the way of its introduction was done through land routes strictly through the movement of vehicles.

Our study reports an established population of the Asian tiger mosquito in the municipality of Prizren in the Northern part of Kosovo.

The ovitraps were selected as a research method due to their high sensitivity to low mosquito density, low price and practical use in the field [43]. A female tiger mosquito can lay eggs in several ovitraps placed in different areas; however, it depends on the sites' attractiveness [44]. Ovitraps can help control the mosquito population by eliminating the eggs, which results in a lower number of mosquitoes [17]. The first *Ae. albopictus* specimen is registered in Zhur, a village close to the border with Albania. This specimen recorded in a garden with many artificial breeding places (container- breeding species) adapted to the temperate climate affected by the Mediterranean climate, channeled from the Adriatic Sea in Albania. The tiger mosquito it have ability to use both natural and artificial containers for larval habitats facilitates its widespread occupation of urban and peri urban environments [30], ensuring a close connection between the species and the human population, increasing the risk of vector-borne diseases in these areas [45].

The other sampling station, Vërmicë, is the closest to Albania's border, which argues the significant presence of eggs in the ovitraps and the large number of adults caught with BG-sentinel trap. Border zones considered key to the introduction of invasive species. The number of cars and other vehicles coming from Albania is vast, so introducing the tiger mosquito through this road has been indisputable. After that, from the border with Albania continue the highways,

Morin-Prizren-Prishtinë it is expected that mosquitoes will spread in other parts of Kosovo, precisely through the land route. Also, the third sampling station in Vlashnje is characterized by the significant presence of used tires, which served as the breeding sides for egg release. In the monitoring station inside the city of Prizren, the number of eggs in ovitraps was high, resulting in an increased number of adults. This finding shows the ability of the tiger mosquito to adapt to the environments where humans live. The recording of *Ae. albopictus* on this part of Kosovo is an important finding, demarcating new boundaries of the distribution range of the species in Europe and indicating the possibility of this species spreading the risk of diseases that it can bring. In the Landovica locality located in the periphery of Prizren, we didn't have any egg of mosquito and based on this, it suggests that the last limit of its spread has been up to this year the city of Prizren. Also, in Suhareka, we didn't have any egg in the ovitraps, considering that this locality was about 41 km away from the Albanian border. However, with almost the same climatic conditions and approximate altitude, the results show that the farthest limit for the spread of the tiger mosquito is isolated only in the municipality of Prizren for this year.

Conclusions

Our results of introducing the tiger mosquito in Kosovo suggest that the Health authorities should establish a national monitoring system to evaluate the spread of the tiger mosquito in other regions of the country and prevent the risk of an invasion. Since community involvement has been very successful in other countries, we suggest that community involvement (citizen scientists) should be highly encouraged for the early detection of an invasive mosquito species.

Our results show the presence of the tiger mosquito in Kosovo for the first time in June, 2020. Actually *Ae. albopictus* is located in the southern part of Kosovo, but with the increased intensity of road transport, it is expected that it will soon spread throughout the country and bring the risk for Aedes-borne disease. Transmission prevention of this species should be regarded as high priority for public health authorities in Kosovo.

Acknowledgments

The work was done within the framework of AIM-COST Action CA17108.

We would like to express our gratitude to Prof. Dr Silvia Bino, Head of the Department of Epidemiology and Control of Infectious Diseases, Institute of Public Health in Tirana, Albania,

for her continuous support of our research and Mihaela Kavran that provided papers for research conducted on Serbia. Ovitraps used in this study were provided by ECDC VectorNet project (framework contract OC/EFSA/AHAW/2013/02-FWC1 funded by the European Food Safety Authority (EFSA) and the European Centre for Disease prevention and Control (ECDC).

We are grateful to Lulzim Alia, geographer from Prishtina, Kosovo, who helps us to prepare the map of the Figure 3 of this manuscript. We also want to thank the community of the village Zhur and other localities in Kosovo for their cooperation to provide information on mosquito's presence and for giving us permission to investigate in their properties during the fieldwork.

References

1. Medlock JM, Hansford KM, Versteirt V, Cull B, Kampen H, Fontenille D, et al. An entomological review of invasive mosquitoes in Europe. Bull Entomol Res. 2015; 105, 637-663.

2. Badieritakis E, Papachristos D, Latinopoulos D, Stefopoulou A, Kolimenakis A, Bithas K, et al. *Aede salbopictus* (Skuse,1895) (Diptera: Culicidae) in Greece: 13 years ofliving with the Asian tiger mosquito. 2018 Parasitol Res.117, 453-460.https://doi.org/10.1007/s00436-017-5721-6PMid:29275504

3. Rosen L. Dengue in Greece in 1927 and 1928 and he pathogenesis of dengue hemorrhagic fever: new data and a different conclusion. Am J Trop Med Hyg 1986; 35:642–653.

4. Mitchell CJ. The role of *Aedes albopictus* as an arboviral vector. *Parassitologia*, 1995 a. **37**, 109-113.

5. Mitchell CJ, Haramis LD, Karabatsos N, et al. Isolation of La Crosse, Cache Valley, and Potosi viruses from Aedes mosquitoes (Diptera: Culicidae) collected at used-tire sites in Illinois during 1994–95. J Med Entomol 1998;35:573–7.

6. Gratz NG. Critical review of the vector status of *Aedes albopictus*. *Medical and Veterinary Entomology*, 2004: **18,** 215-227.

7. Tilston N, Skelly C, and Weinstein P. Pan-European Chikungunya surveillance: Designing risk stratified surveillance zones. Int J Heath Geog 2008; 8:61.

8. World Health Organization (WHO). Comprehensive Guidelines for Prevention and Control of Dengue and Dengue Haemorrhagic Fever. WHO Regional Publication, South-East Asia Series 1999; 29:196 pp.
9. Rezza G, Nicoletti L, Angelini R, Romi R, Finarelli A.C, Panning M. Infection with chikungunya virus in Italy: an outbreak in a temperate region. Lancet. 2007; 370:1840–6
10. Gossner C M, Ducheyne E, & Schaffner F. Increased risk for autochthonous vector-borne infections transmitted by Aedes albopictus in continental Europe. *Eurosurveillance*, 2018: *23*(24).
11. ECDC, 2019 https://www.ecdc.europa.eu/en/news-events/epidemiological-update-third-case-locally-acquired-zika-virus-disease-hyeres-france

12. Jourdain F, Roiz D, de Valk H, Noël H, L'Ambert G, Franke F, et al. From importation to autochthonous transmission: Drivers of chikungunya and dengue emergence in a temperate area. *PLoS neglected tropical diseases*, 2020; *14*(5), e0008320. https://doi.org/10.1371/journal.pntd.0008320

13. Vermeulen TD, Reimerink J, Reusken C, Giron S, & de Vries PJ. Autochthonous dengue in two Dutch tourists visiting Département Var, southern France, July 2020. *Euro surveillance : bulletin Europeen sur les maladies transmissibles = European communicable disease bulletin*, 2020; *25*(39), 2001670. https://doi.org/10.2807/1560-7917.ES.2020.25.39.2001670
14. Adhami J, and Murati N. Prani e mushkonjës *Aedes albopictus* në Shqipëri. Rev Mjekesore. 1987; l:13–6.
15. Velo E, Kadriaj P, Mersini K, Shukullari A, Manxhari B, Simaku A, et al. Enhancement of *Aedes albopictus* collection by ovitrap and sticky adult trap. Parasites & Vectors. 2016; 9:223 https://doi.org/10.1186/ s13071-016-1501-x PMID: 27102015
16. Petric D, Zgomba M, Ignjatovic Cupina A, Pajovic I, Merdic E, Boca I, et al. Invasion of the *Stegomyia albopicta* to a part of Europe. *Abstracts of the SOVE 15th European Meeting, Serres, Greece*, 2006; p58
17. Cvetkovikj A, Djadjovski I, Krstevski K, Popova Z, Rashikj L, Atanasova K, et al. New Records of the Asian Tiger Mosquito (*Aedes albopictus*) in North Macedonia. Macedonian Veterinary Review. Mac Vet Rev 2020; 43 (2): i-v

18. Petrić D, Pajović I, Ćupina A, and Zgomba M. *Aedes albopictus* in Entomofauna of Yugoslavia. Symposia of Serbian Entomologist's 2001, Entomological Society of Serbia, Goc. 26–29 September 2001.

19. Petrić D, Zgomba M, Ignjatović CA, Marinković D, Bellini R, Schaffner F, et al. Invasive mosquito species in Europe and Serbia, 1979-2011. International Symposium on Current Trends in Plant Protection. 2012.

20. ECDC 2014, http://ecdc.europa.eu/en/healthtopics/vectors/vector-maps/Pages/VBORNET_maps.aspx

21. Knudsen AB, Romi R, and Majori G. Occurrence and spread in Italy of *Aedes albopictus*, with implications for its introduction into other parts of Europe. J Am Mosq Control Assoc 1996; 2:177–183

22. Medlock JM, Avenell D, Barrass I, Leach S. Analysis of the potential for survival and seasonal activity of *Aedes albopictus* 760 OTER ET AL. (Diptera: Culicidae) in the United Kingdom. J Vector Ecol 2006; 31:292–304.

23. Benedict MQ, Levine R, Hawley WA, & Lounibos P. Spread of the Tiger: Global Risk of Invasion by the Mosquito *Aedes albopictus* Vector Borne Zoonotic Dis. 2007; 7(1): 76–85. (https://doi.org/10.1089/vbz.2006.0562) PMID: 17417960

24. Reiter P, and Sprenger D. The used tire trade: a mechanism for the worldwide dispersal of container breeding mosquitoes. J Am Mosq Control Assoc. 1987; 3: 494–501.

25. Linthicum KJ, Kramer VL, Madon MB, and Fujioka K. Introduction and potential establishment of *Aedes albopictus* in California in 2001. J Am Mosq Control Assoc 2003; 19:301–308.

26. Scholte EJ, Dijkstra E, Blok H, De Vries A, Takken W, Hofhuis A. Accidental importation of the mosquito *Aedes albopictus* into the Netherlands: a survey of mosquito distribution and the presence of dengue virus. Med Vet Entomol, 2008; 22:352–358.

27. Muja-Bajraktari N, Zhushi-Etemi F, Dikolli-Velo E, Kadriaj P, and Gunay F. The composition, diversity, and distribution of mosquito fauna (Diptera: Culicidae) in Kosovo. *Journal of Vector Ecology*. 2019; 44 (1): 94-104.

28. Bellini R, Michaelaki A, Petrić D, Schaffner F, Alten B, Angelini A, et al. Practical management plan for invasive mosquito species in Europe: I. Asian tiger mosquito (*Aedes*

albopictus), Travel Medicine and Infectious Disease. 2020; https://doi.org/10.1016/j.tmaid.2020.101691

29. Gunay F, Picard M, and Robert V. MosKeyTool, an interactive identification key for mosquitoes of Euro-Mediterranean. 2018 Version 2.1.www.medilabsecure.com/moskeytool. Last update:01/08/2018.

30. Hawley W. The biology of *Aedes albopictus*. *J. Am. Mosq. Contr. Assoc.*, 1988 (Suppl.) 4, 1-39).

31. Sabatini A, Ranieri V, Trovato G, and Coluzzi M. *Aedes albopictus* in Italia e possible diffusione de lla specienell'area mediterranea.*Parassitologia*, 1990: **32**, 301-304.

32. Schaffner F, and Karch S. Première observationd'*Aedes albopictus* (Skuse, 1894), en France métropolitaine. *Sciences de la Vie/Life Sciences*, 2000 : **323**(4), 373-375.

33. Schaffner F, Bouletreau B, Guillet B, Guilloteau J, and Karch S. *Aedes albopictus* (Skuse, 1894), established in metropolitan France.*European Mosquito Bulletin*, 2001: **9**, 1-3.

34. Roiz D, Eritja R, Escosa R, Lucientes J, Marques E, Melero-Alcibar R, et al. Spreading of *Aedes albopictus* in Spain. *Abstracts of the SOVE 15thEuropean Meeting, Serres, Greece*, 2006: p60.

35. Schaffner F, Van Bortel W, and Coosemans M. First record of *Aedes* (*Stegomyia*) *albopictus* in Belgium. *Journal of the American Mosquito Control Association*, 2004: **20**(2), 201-203.

36. Flacio E, Patocchi N, Peduzzi R, and Luethy P. Strategies to stop the establishment of *Aedes albopictus* in Switzerland. Abstracts of the SOVE15th European Meeting, Serres,Greece, 2006, p57.

37. Samanidou-Voyadjoglou A, Patsoula E, Spanakos G, and Vakalis NC. Confirmation of *Aedes albopictus* (Skuse) (Diptera: Culicidae) in Greece. European Mosquito Bulletin, 2005:19, 10-11. Journal of the European Mosquito Control Association ISSN1460-6127

38. Becker N, Petric D, Zgomba M, Boase C, Dahl C, Lane J, at al. Mosquitoes and Their Control. Kluwer Academic/Plenum Publishers, New York. 2003. ISBN 0-306-47360-7. pp.498.

39. Schaffner F. Updated data on non-indigenous invasive mosquitoes in Europe, with special reference to France. *Abstracts of the SOVE 15thEuropean Meeting, Serres, Greece*, 2006 p56.

40. Buhagiar JA. A second record of Aedes (Stegomyia) albopictus (Diptera: Culicidae) in Malta. Eur Mosq Bull. 2009; 27 : 65–67.

41. Bengoa M, Delacour-Estrella S, Barceló C, Paredes-Esquivel C, Leza M, and Lucientes J. First record of Aedes albopictus (Skuse, 1894) (Diptera; Culicidae) from Minorca (Balearic Islands, Spain). J Eur Mosq Control Ass. 2016;34:5–9.

42. Eritja R, Palmer JRB, Sanpera-Calbet DRI, and Bartumeus F. Direct evidence of Adult *Aedes albopictus* Dispersal by Car. Scientific Reports 2017 | 7: 14399 | DOI:10.1038/s41598-017-12652-5. Article number: 14399

43. Carrieri M, Albieri A, Angelini P, Baldacchini F, Venturelli C, Zeo S.M, et al. Surveillance of the chikungunya vector *Aedes albopictus* (Skuse) in Emilia-Romagna (northern Italy): Organizational and technical aspects of a largescale monitoring system. J Vector Ecol. 2011; 36, 108-116.

44. Manica M, Filipponi F, D'Alessandro A, Screti A, Neteler M, Rosà R, et al. Spatial and temporal hot spots of *Aedes albopictus* abundance inside and outside a South European metropolitan area. PLoSNegl Trop Dis. 2016; 10, 1-17. PMid:27333276 PMCid: PMC4917172

45. Takken W, and Knols B Gj. Emerging Pests and Vector-Borne Diseases in Europe, vol. 1. The Netherlands: Wageningen Academic Publishers, 2007:499 pp.

 Click here to access/download;Figure;Fig1.png

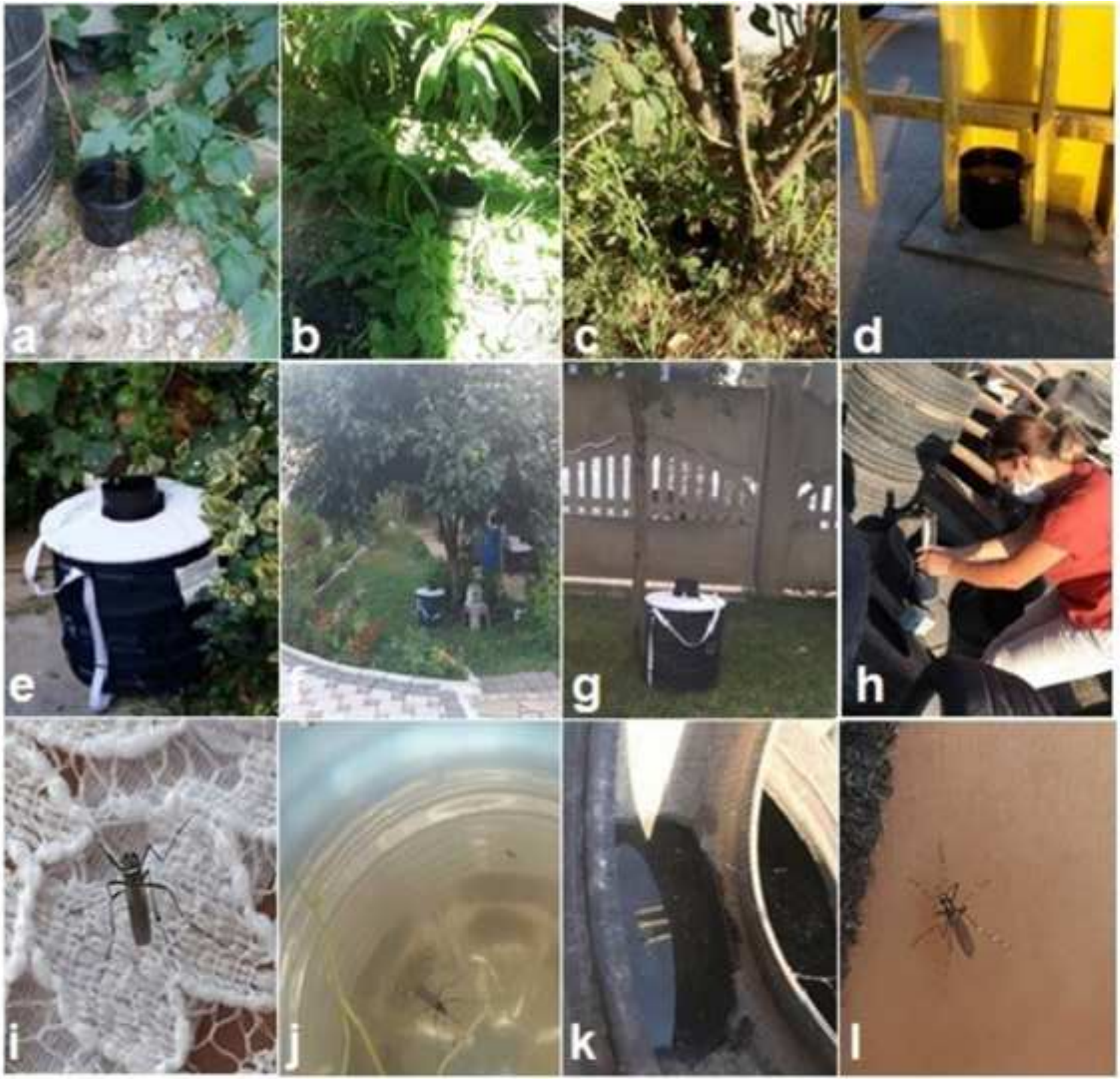

Figure

Click here to access/download;Figure;Fig2.jpeg

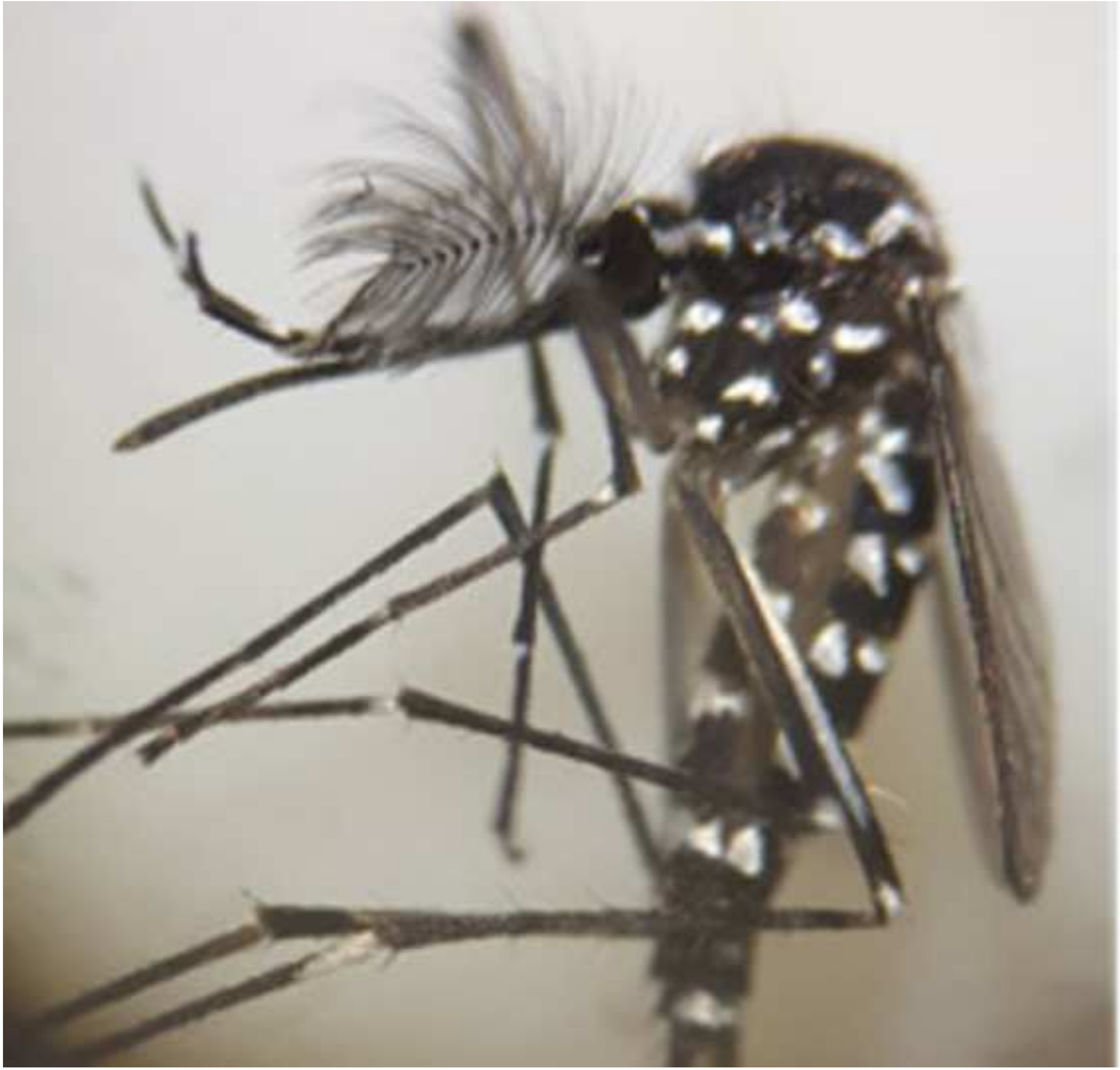

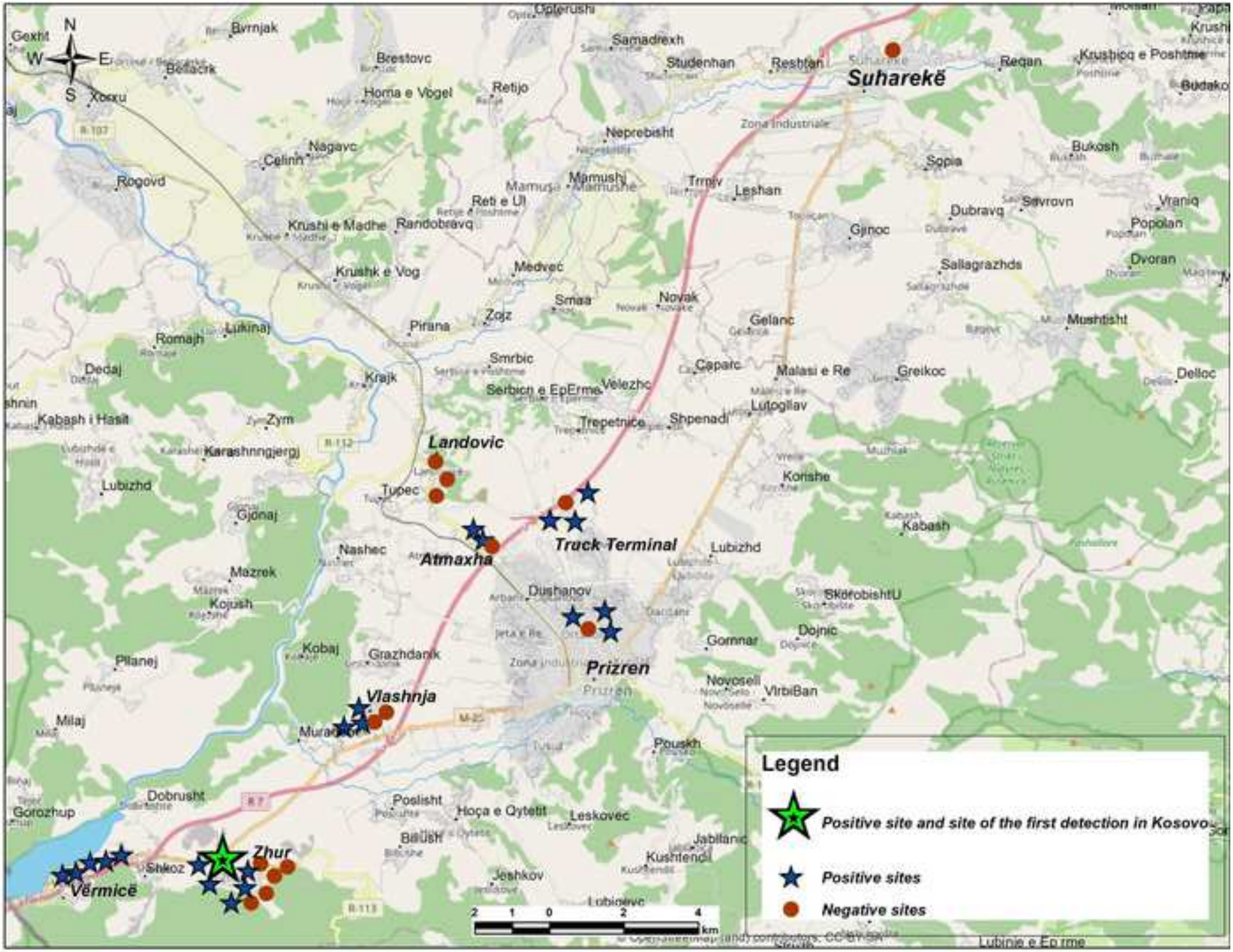
Suharekë
Landovic
Truck Terminal
Atmaxha
Prizren
Vlashnja
Zhur
Vërmicë
Legend
Positive site and site of the first detection in Kosovo
Positive sites
Negative sites
km
