# Supplementary material for: The Asian tiger mosquito Aedes albopictus (Skuse) in Kosovo: First record
Source: PLoS One. 2022 Mar 15;17(3):e0264300. doi: 10.1371/journal.pone.0264300 (PMC8923454; doi:10.1371/journal.pone.0264300)
Supplement: Attachment — Submitted filename: Response to Reviewers.docx [file pone.0264300.s003.docx]

Response to Reviewers

Dear reviewers,

Thank you very much for the given comments. We find them very useful and tried to answer to each of them.

Answer to Journal requirements

1. Please ensure that your manuscript meets PLOS ONE's style requirements, including those for file naming. The PLOS ONE style templates can be found at 
<https://journals.plos.org/plosone/s/file?id=wjVg/PLOSOne_formatting_sample_main_body.pdf>and 
<https://journals.plos.org/plosone/s/file?id=ba62/PLOSOne_formatting_sample_title_authors_affiliations.pdf>

Answer: Manuscript is corrected according to the Journal requirements

2. In your Methods section, please provide additional information regarding the permits you obtained for the work. Please ensure you have included the full name of the authority that approved the field site access and, if no permits were required, a brief statement explaining why.

Answer: Since the village Zhur where the first specimen of *Aedes albopictus* was recorded does not belong to a protected area, there was no need to ask for the permit to conduct our research. There was no need for permit to carry out the study also for the other localities. Citizens were very collaborative and allowed us to collect in their properties.

3. We note that Figure 3 in your submission contain [map/satellite] images which may be copyrighted. All PLOS content is published under the Creative Commons Attribution License (CC BY 4.0), which means that the manuscript, images, and Supporting Information files will be freely available online, and any third party is permitted to access, download, copy, distribute, and use these materials in any way, even commercially, with proper attribution. For these reasons, we cannot publish previously copyrighted maps or satellite images created using proprietary data, such as Google software (Google Maps, Street View, and Earth). For more information, see our copyright guidelines: <http://journals.plos.org/plosone/s/licenses-and-copyright>.

Answer: A new map has been designed by another author for the purpose to be used in our Manuscript according to the PLOS ONE policy.

The author of the new map, Mr. Kreshnik Morina (Geodesic) submitted a confirmation about the authorship and granted the permission to the authors of the Manuscript to use the content of the map for publication. He also explained that the general administrative maps were extracted from the Natural Earth (a public domain map dataset) <https://www.naturalearthdata.com/> which is free to use in any type of project and then modified subsequently according to the information presented in the map, using Q-GIS version 3.18 (an open source GIS program).

4. We note that Figure 1 includes an image of a [patient / participant / in the study]. 
As per the PLOS ONE policy (<http://journals.plos.org/plosone/s/submission-guidelines#loc-human-subjects-research>) on papers that include identifying, or potentially identifying, information, the individual(s) or parent(s)/guardian(s) must be informed of the terms of the PLOS open-access (CC-BY) license and provide specific permission for publication of these details under the terms of this license. Please download the Consent Form for Publication in a PLOS Journal (<http://journals.plos.org/plosone/s/file?id=8ce6/plos-consent-form-english.pdf>). The signed consent form should not be submitted with the manuscript, but should be securely filed in the individual's case notes. Please amend the methods section and ethics statement of the manuscript to explicitly state that the patient/participant has provided consent for publication: “The individual in this manuscript has given written informed consent (as outlined in PLOS consent form) to publish these case details”.

If you are unable to obtain consent from the subject of the photograph, you will need to remove the figure and any other textual identifying information or case descriptions for this individual.

Answer: In the image of the figure is the first author of the manuscript during the research in the field. She included her photo in the Manuscript.

5. Please review your reference list to ensure that it is complete and correct. If you have cited papers that have been retracted, please include the rationale for doing so in the manuscript text, or remove these references and replace them with relevant current references. Any changes to the reference list should be mentioned in the rebuttal letter that accompanies your revised manuscript. If you need to cite a retracted article, indicate the article’s retracted status in the References list and also include a citation and full reference for the retraction notice.

Answer: The references are checked carefully.

Reviewers' comments:

Reviewer's Responses to Questions

**Comments to the Author**

1. Is the manuscript technically sound, and do the data support the conclusions?

The manuscript must describe a technically sound piece of scientific research with data that supports the conclusions. Experiments must have been conducted rigorously, with appropriate controls, replication, and sample sizes. The conclusions must be drawn appropriately based on the data presented.

Reviewer #1: Yes

Reviewer #2: Yes

2. Has the statistical analysis been performed appropriately and rigorously?

Reviewer #1: I Don't Know

Reviewer #2: N/A

3. Have the authors made all data underlying the findings in their manuscript fully available?

The [PLOS Data policy](http://www.plosone.org/static/policies.action#sharing) requires authors to make all data underlying the findings described in their manuscript fully available without restriction, with rare exception (please refer to the Data Availability Statement in the manuscript PDF file). The data should be provided as part of the manuscript or its supporting information, or deposited to a public repository. For example, in addition to summary statistics, the data points behind means, medians and variance measures should be available. If there are restrictions on publicly sharing data—e.g. participant privacy or use of data from a third party—those must be specified.

Reviewer #1: Yes

Reviewer #2: Yes

4. Is the manuscript presented in an intelligible fashion and written in standard English?

PLOS ONE does not copyedit accepted manuscripts, so the language in submitted articles must be clear, correct, and unambiguous. Any typographical or grammatical errors should be corrected at revision, so please note any specific errors here.

Reviewer #1: No

Reviewer #2: Yes

5. Review Comments to the Author

Please use the space provided to explain your answers to the questions above. You may also include additional comments for the author, including concerns about dual publication, research ethics, or publication ethics. (Please upload your review as an attachment if it exceeds 20,000 characters)

Reviewer #1: The authors describe the first records of tiger mosquitoes in Kosovo. The manuscript is of high relevance in the field and provides novel information.

Major comment: English language editing is needed.

Answer: The English editing is done as suggested.

Minor comments:
entire document – e.g. Line 31: Change Aedes to Ae. when needed (not at first mentioning and at beginning of sentences)

Answer: It is changed as suggested

The definition “invasive” should be used with care. It has not proven to outcompete native mosquitoes in Kosovo. It is recommended to change to “potential invasive”

Answer: It is corrected. In the revised text is used potential invasive.

Line 64: Mosquitoes transmit pathogens and not diseases.

Answer: In the line 61 it is stated that *Ae. albopictus*has potential to transmit 22 arboviruses-pathogens, of the family Flaviviridae

Line 82: Dracaena in italics

Answer: it is corrected

Line 109: The link to BG is not necessary. It is better to mention BG-sentinel generation 2. Moreover it is not mentioned if CO2 and lure were used. Info is give in line 139 – recommended to move to line 109
Answer: It is corrected as suggested by reviewer.
Line 182: s.l. not in italics.

Answer: Corrected.
Line 180-190: Many countries are missing – if all European countries where Ae. albopictus was reported are planned to be listed

Answer: Other European countries where *Ae.albopictus* is reported are added.

Mosquito alert is mentioned but no data shown. Maybe discuss that this app might increase the number of reports (the reviewer has seen tiger mosquitoes from the Kosovo when specifying for Mosquito Alert).

Answer: The first author of the Manuscript used Mosquito Alert to report first record of *Ae. albipictus* in her country.

Reviewer #2: The manuscript is well written and provides interesting findings from Kosovo. There are some issues however to be addressed and most of them are commented directly in the manuscript pdf document.

The abstract is somehow too long, and it would be nice to be shortened.

Answer: Abstract is shortened as suggested.

In Introduction or somewhere in the Discussion section it would be advisable to give an overview of other invasive insects known from Kosovo, so that the paper is put also in the context of broader invasion challenge.

Answer: As suggested by the reviewer, an overview of other insect invasive species reported from Kosovo is given in the Discussion.

English must be improved as well, although most of the linguistic corrections are done inside the document. I suggest publishing the paper after these revisions.

Answer: The language corrections are done.

Beside the correction based on the reviewers’ comments and suggestions, some other minor correction are done in the Manuscript aiming to improve its quality.

6. PLOS authors have the option to publish the peer review history of their article ([what does this mean?](https://journals.plos.org/plosone/s/editorial-and-peer-review-process#loc-peer-review-history)). If published, this will include your full peer review and any attached files.


If you choose “no”, your identity will remain anonymous but your review may still be made public.


**Do you want your identity to be public for this peer review?** For information about this choice, including consent withdrawal, please see our [Privacy Policy](https://www.plos.org/privacy-policy).

Reviewer #1: No

Reviewer #2: **Yes:** HalilIbrahimi

[NOTE: If reviewer comments were submitted as an attachment file, they will be attached to this email and accessible via the submission site. Please log into your account, locate the manuscript record, and check for the action link "View Attachments". If this link does not appear, there are no attachment files.]

While revising your submission, please upload your figure files to the Preflight Analysis and Conversion Engine (PACE) digital diagnostic tool, <https://pacev2.apexcovantage.com/>. PACE helps ensure that figures meet PLOS requirements. To use PACE, you must first register as a user. Registration is free. Then, login and navigate to the UPLOAD tab, where you will find detailed instructions on how to use the tool. If you encounter any issues or have any questions when using PACE, please email PLOS at [figures@plos.org](mailto:figures@plos.org). Please note that Supporting Information files do not need this step.
